# Usefulness of DNA Methylation Levels in *COASY* and *SPINT1* Gene Promoter Regions as Biomarkers in Diagnosis of Alzheimer’s Disease and Amnestic Mild Cognitive Impairment

**DOI:** 10.1371/journal.pone.0168816

**Published:** 2016-12-19

**Authors:** Nobuyuki Kobayashi, Shunichiro Shinagawa, Tomoyuki Nagata, Kazuya Shimada, Nobuto Shibata, Tohru Ohnuma, Koji Kasanuki, Heii Arai, Hisashi Yamada, Kazuhiko Nakayama, Kazuhiro Kondo

**Affiliations:** 1 Department of Virology, The Jikei University School of Medicine, Tokyo, Japan; 2 Department of Psychiatry, The Jikei University School of Medicine, Tokyo, Japan; 3 Division of Molecular Genetics, The Jikei University School of Medicine, Tokyo, Japan; 4 Department of Psychiatry, Juntendo University School of Medicine, Tokyo, Japan; Universitatsklinikum Leipzig, GERMANY

## Abstract

In order to conduct early therapeutic interventions for Alzheimer’s disease (AD), convenient, early diagnosis markers are required. We previously reported that changes in DNA methylation levels were associated with amnestic mild cognitive impairment (aMCI) and AD. As the results suggested changes in DNA methylation levels in the *COASY* and *SPINT1* gene promoter regions, in the present study we examined DNA methylation in these regions in normal controls (NCs, n = 30), aMCI subjects (n = 28) and AD subjects (n = 30) using methylation-sensitive high resolution melting (MS-HRM) analysis. The results indicated that DNA methylation in the two regions was significantly increased in AD and aMCI as compared to NCs (*P* < 0.0001, *P* < 0.0001, ANOVA). Further analysis suggested that DNA methylation in the *COASY* gene promoter region in particular could be a high sensitivity, high specificity diagnosis biomarker (*COASY*: sensitivity 96.6%, specificity 96.7%; *SPINT1*: sensitivity 63.8%, specificity 83.3%). DNA methylation in the *COASY* promoter region was associated with CDR Scale Sum of Boxes (CDR-SB), an indicator of dementia severity. In the *SPINT1* promoter region, DNA methylation was negatively associated with age in NCs and elevated in aMCI and AD subjects positive for antibodies to Herpes simplex virus type 1 (HSV-1). These findings suggested that changes in DNA methylation in the *COASY* and *SPINT1* promoter regions are influenced by various factors. In conclusion, DNA methylation levels in the *COASY* and *SPINT1* promoter regions were considered to potentially be a convenient and useful biomarker for diagnosis of AD and aMCI.

## Introduction

Alzheimer’s disease (AD) is a neurodegenerative disease characterized by cognitive dysfunction, of which memory impairment is a typical feature, and the disease goes through the prior stage of amnestic mild cognitive impairment (aMCI) before onset [1]. The pathophysiological process of AD is thought to begin many years before diagnosis [2], and though therapeutic interventions are desirable at this stage, diagnosis is difficult. Therefore, we need to develop biomarkers that can predict progression to aMCI and AD.

Some of the known procedures for the early diagnosis of AD are amyloid imaging using positron-emission tomography (PET) [3], and measurement of amyloidβ (Aβ)_1-42_ or tau in cerebrospinal fluid (CSF) [4, 5]. However, lacking specificity and convenience, and being invasive, they have shortcomings as diagnosis tools. So, there is a great need to develop convenient new blood biomarkers.

In recent years, changes in DNA methylation levels for several genes in the AD brain have been reported [6–9]. In our previous research, we investigated blood DNA genome wide for candidate loci with variation in methylation levels in age- and sex-matched normal controls (NCs), aMCI and AD patients. We demonstrated that changes in methylation levels were occurring in the *NCAPH2/LMF2* promoter region in AD and aMCI [10] and our findings suggested that there were changes in methylation levels in many other regions besides this one. Screening according to the conditions of progressive increase in DNA methylation going from NC to aMCI to AD, registered gene with UCSC accession number, and DNA methylation occurring in promoter region in CpG island produced 10 candidate loci in addition to the *NCAPH2/LMF2* promoter region [10].

Among them, a correlation between mini-mental state examination (MMSE) and DNA methylation level was noted for areas in the *COASY*, *SPINT1* and *RERG* gene promoter regions. As a detailed examination of methylation levels in these 3 regions had not been previously conducted, in the present research, we measured them by methylation-sensitive high resolution melting (MS-HRM) analysis [11]. Compared to pyrosequencing, MS-HRM is lower in cost but produces similar results [12].

Aging, chronic inflammation and virus infections are generally known causes of DNA methylation [13]. In recent years many studies, including one of our own, have reported associations between infectious burden, notably that due to Herpes simplex virus 1 (HSV-1), and AD [14–17]. Also, *APOE* allele 4 is known to be the major genetic risk factor for AD [18]. Therefore, in the present study, we investigated associations between clinical background factors, including HSV-1 infection and *APOE* genotype, and DNA methylation.

In view of the foregoing, the objectives of the present study were to clarify whether DNA methylation could be a useful diagnosis biomarker for aMCI and AD and explore associations between it and background factors.

## Materials and Methods

### Ethics statement

The study was approved by the Ethics Committee of the Jikei University School of Medicine and Juntendo University School of Medicine, and written informed consent was obtained from all subjects. For participants whose capacity to consent was compromised, caregivers who were the spouse or a relative within the second degree consented on their behalf. For patients with aMCI or AD who were able to sign the consent form, if there was any possibility of them forgetting that they had consented to participation, written informed consent was obtained from both patients and caregivers.

### Subjects

The subjects of the present study were the same as those in our previous study [10] and their characteristics are given in Table 1. The details of their selection are given in the following. From among consecutive memory clinic outpatients visiting the Jikei University Hospital (Tokyo) or the Jikei University Kashiwa Hospital (Kashiwa City, Chiba Prefecture), 30 patients with AD and 28 patients with aMCI were enrolled. The 30 NCs were recruited from Juntendo University Hospital (Tokyo) [10, 19, 20]. Each group included the subjects whose samples were analyzed by the Illumina Infinium HD Methylation Assay in our previous study [10]. AD was diagnosed based on the US National Institute of Neurological and Communicative Disorders and Stroke and the Alzheimer's Disease and Related Disorders Association (NINCDS-ADRDA) criteria, and aMCI by the criteria defined by Peterson [21, 22]. aMCI included both amnestic MCI-single domain- and MCI-multiple domain-type subjects. The MMSE was administered to AD and aMCI patients by a clinical psychologist [23, 24]. To determine the severity of each patient’s dementia, geriatric psychiatrists determined global CDR scores while interviewing each patient’s caregiver [25]. The neuropsychiatric symptoms for AD were assessed based on information from a structured interview with each patient’s caregiver by geriatric psychiatrists using the behavioral pathology in Alzheimer’s disease (BEHAVE-AD) scale [26]. NCs were those who did not satisfy the clinical criteria for AD or MCI, but if they had past history of treatment for other psychiatric disorders, they were excluded. No patients with acute infections were included in the subjects and there were no deviations in differential white blood cell counts among them. Genomic DNA was extracted from white blood cells using a standard method [19, 20].

For aMCI and AD subjects, plasma was collected from whole blood, centrifuged at 2000 rpm for 20 minutes and stored at -80°C until analysis. Plasma samples were not collected for NCs.

**Table 1:** Subject characteristics (mean ± S.E.M.)

	NC (n = 30)	MCI (n = 28)	AD (n = 30)	*P*
Age (years)	70.5 ± 1.0	72.0 ± 0.9	71.8 ± 0.9	0.529
Female: male (%)	60.0: 40.0	53.6: 46.4	53.3: 46.7	0.842
Duration of disease (months)	-	26.5 ± 4.4	28.2 ± 3.5	0.774
Age at onset (years)	-	69.8 ± 0.9	69.4 ± 1.0	0.789
MMSE score	-	27.2 ± 0.4	18.5 ± 1.0	0.000 ****
CDR-SB	-	2.2 ± 0.2	6.1 ± 0.4	0.000 ****
BEHAVE-AD	-	3.0 ± 0.5	5.4 ± 0.8	0.018 *
*APOE* ε2/ε2 No. (%)	1 (3.3)	0 (0)	0 (0)	
*APOE* ε2/ε3 No. (%)	1 (3.3)	1 (3.6)	0 (0)	
*APOE* ε3/ε3 No. (%)	17 (56.7)	17 (60.7)	11 (36.7)	
*APOE* ε3/ε4 No. (%)	11 (36.7)	8 (28.6)	13 (43.3)	
*APOE* ε4/ε4 No. (%)	0 (0)	2 (7.1)	6 (20)	
*APOE* ε2 No. (%)	3 (5.0)	1 (1.8)	0 (0)	0.551
*APOE* ε3 No. (%)	46 (76.7)	43 (76.8)	35 (58.3)	0.144
*APOE* ε4 No. (%)	11 (18.3)	12 (21.4)	25 (41.7)	0.036 *
Anti-HSV-1 IgG positive No. (%)	-	22 (81.5) ^a^	17 (60.7) ^b^	0.09

Age was analyzed by the Kruskal-Wallis test. Sex ratio, *APOE* allele frequencies and anti-HSV-1 IgG positive rate were analyzed using the chi-squared test. The other parameters were analyzed by Welch's t-test.

* *P* < 0.05

**** *P* < 0.0001

^a^ n = 27

^b^ n = 28.

### Anti-HSV-1 antibody titer

We used an HSV-1 IgG ELISA Kit (Phoenix Pharmaceuticals, Inc.). The anti-HSV-1 antibody titer was measured according to the attached protocol. A TriStar LB941-vTi Microplate Reader (Berthold Technologies) was used to measure optical density (450 nm) after the color reaction. Following the attached protocol, the anti-HSV-1 antibody titer was expressed as an HSV-1 IgG index by comparing the patient sample optical density and the cut-off calibrator optical density. A sample was defined as being positive for anti-HSV-1 IgG antibody if the HSV-1 IgG Index was above 1.0.

### MS-HRM

500 ng of each genomic DNA sample was bisulfite-converted using EpiTect Plus DNA Bisulfite Kit (Qiagen, Inc.) and purified. EpiTect Control DNA, methylated (Qiagen, Inc.) was used as the completely methylated control DNA and EpiTect Control DNA, unmethlyated (Qiagen, Inc.) as unmethylated DNA. Various mixtures of them were made to produce a calibration curve for methylation rates of 100%, 75%, 50%, 25%, 5% and 0%.

Amplifications were performed in a total volume of 20 μl containing 10 μl of 2x MeltDoctor HRM Master Mix (Applied Biosystems), 0.12 μl of 50 μM forward primer, 0.12 μl of 50 μM reverse primer, 2 μl of the bisulfited DNA and 7.76 μl of PCR-grade water. The thermal profile was 95°C for 10 min, 50 cycles of 95°C for 15 sec, and 60°C for 60 sec. The primers were as follows: *COASY* forward primer, 5′-GATTATGGGATAGGAGAAGTGTT-3′; *COASY* reverse primer, 5′-CCTAATCCAAAATCCCTCTTAC-3′; *SPINT1* forward primer, 5′-TGTATATATAAGATGAGGAGGGGT-3′; *SPINT1* reverse primer, 5′-ACCCTCAAAAAATCATTTCCATTTC-3′; *RERG* forward primer, 5′-GTAGTTTTTTTGGTTTGGAGAGT-3′; *RERG* reverse primer, 5′-CTCAACAAATATCTTACAAAAATAAACA-3′.

MS-HRM was performed with the Applied Biosystems QuantStudio 12K Flex Real-Time PCR System (Applied Biosystems).

### Statistical Analysis

The Shapiro-Wilk W test was used to test the normality of data. One-way ANOVA was used with Scheffe's test post hoc to compare parameters among aMCI, AD, and NC subjects, other than age, gender, *APOE* genotype and anti-HSV-1 IgG positive rate. Age was compared using the Kruskal-Wallis test. Gender, *APOE* genotype and anti-HSV-1 IgG positive rate were compared using the chi-squared test. The Welch’s t-test was used for comparisons between the aMCI and AD groups. Spearman's rank correlation coefficients were used to investigate correlations between individual background characteristics and DNA methylation levels. Student’s t test was used for 2-group comparisons of DNA methylation levels with gender, *APOE* ε4 carrier or HSV-1 carrier. Correlations between the Illumina Infinium HD Methylation Assay and MS-HRM assay were examined using Pearson correlation coefficients. *P* < 0.05 was considered statistically significant.

Statistical analysis was conducted using SPSS Statistics 21 for Windows (IBM) and Prism 6 for Mac OS X (GraphPad Software Inc.).

## Results

### Patient Characteristics

According to the Shapiro-Wilk W test, age of AD subjects, duration of disease for aMCI and AD, age of aMCI onset, AD MMSE score, AD CDR Scale Sum of Boxes (CDR-SB) score, and aMCI and AD BEHAVE-AD scores were not normally distributed. There were no significant differences among the NC, aMCI, and AD groups regarding age and gender, and none were observed for duration of disease and age at onset between the aMCI and AD groups. However, MMSE, CDR-SB and BEHAVE-AD scores were significantly different between the AD group and the aMCI group, and there was a high prevalence of *APOE* allele 4 in the AD group. The *APOE* genotyping data used were from our previous study [10]. There was no difference in anti-HSV-1 antibody positive rate between the aMCI and AD groups (Table 1).

### PCR Amplification

**Fig 1:**
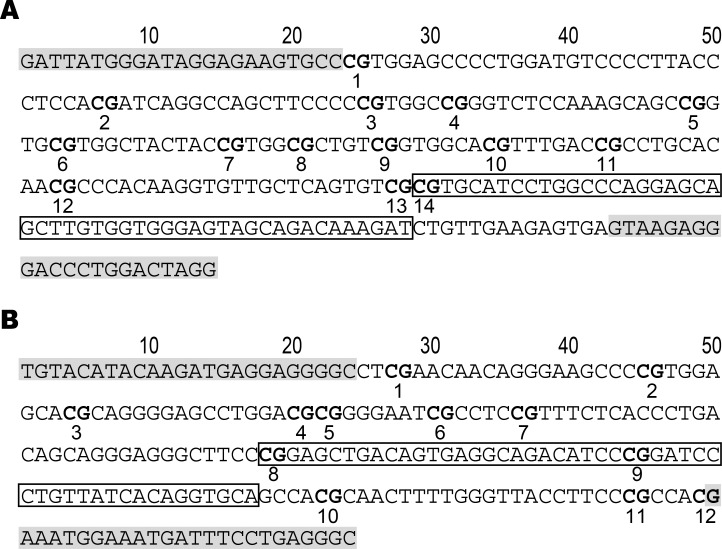
Part of Sequences of *COASY* and *SPINT1* Promoter Regions. The open box area indicates the source sequence of Target id: cg01756799 in the *COASY* promoter region (A) and that of Target id: cg09898695 in the *SPINT1* (B) promoter region. The gray areas indicate the loci of primers.

The size of the *COASY* gene promoter region PCR product was 264 bp and included 14 CpG (Fig 1A). The size of the *SPINT1* gene promoter region PCR product was 224 bp and included 12 CpG (Fig 1B). Methylation in the *RERG* gene promoter region could not be measured because of insufficient PCR amplification. Based on the primer design, the 330 bp PCR band should have appeared after electrophoresis but it was not observable and could not be assessed. Also, in the PCR amplified *COASY* and *SPINT1* gene promoter regions, among the database single nucleotide polymorphisms (dbSNPs) present, there were none that have been determined to be clinical significant SNPs.

### *COASY*, *SPINT1* gene promoter region DNA methylation levels in NC, aMCI and AD subjects

**Fig 2:**
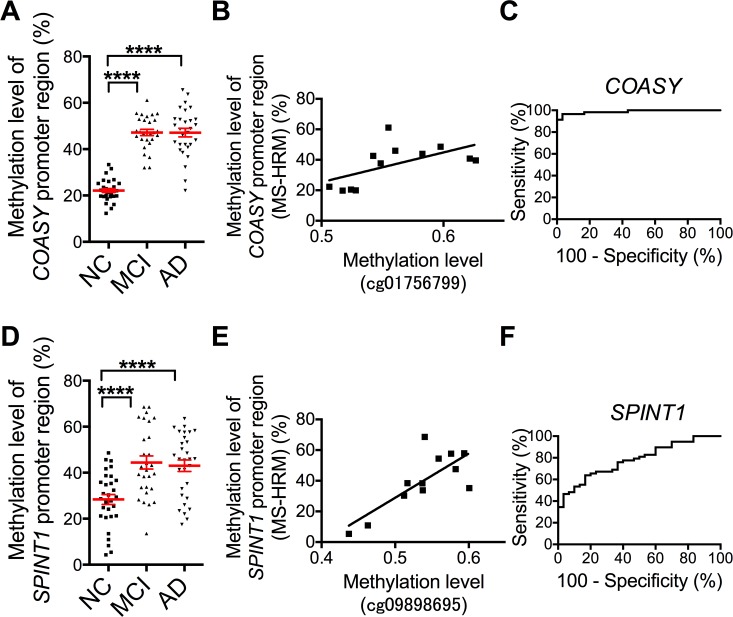
Comparison of Methylation Levels in NC, aMCI and AD groups. For *COASY* gene promoter region: DNA methylation levels in the 3 groups by MS-HRM (A), Correlation between measurement results from MS-HRM and Illumina Infinium HD Methylation Assay (B), receiver operating characteristic (ROC) curve for diagnosis of aMCI and AD (C). For SPINT1 gene promoter region: DNA methylation levels in the 3 groups by MS-HRM (D), Correlation between measurement results from MS-HRM and Illumina Infinium HD Methylation Assay (E), receiver operating characteristic (ROC) curve for diagnosis of aMCI and AD (F). Red horizontal lines are means, error bars indicate S.E.M. ****P < 0.0001; one-way ANOVA with Scheffe’s test.

According to the Shapiro-Wilk W test, *COASY* gene promoter DNA methylation levels followed a normal distribution. Methylation levels in the *COASY* gene promoter region were higher in the aMCI and AD groups as compared with NCs (NC, 22.1 ± 0.9; aMCI, 47.2 ± 1.3; AD, 47.1 ± 1.8% [mean ± S.E.M.]). There was no difference between the aMCI and AD groups (Fig 2A). Our previously published results for 12 specimens also measured by the Illumina Infinium HD Methylation Assay [10] were positively correlated with those from MS-HRM (*r* = 0.86, *P* = 0.000****, ****P < 0.0001; Pearson correlation coefficients) (Fig 2B).

According to the Shapiro-Wilk W test, *SPINT1* gene promoter DNA methylation levels followed a normal distribution. Methylation levels in the *SPINT1* gene promoter region were also higher in the aMCI and AD groups as compared with NCs (NC, 28.4 ± 2.2; aMCI, 44.4 ± 2.9; AD, 43.0 ± 2.5% [mean ± S.E.M.]). There was also no difference between the aMCI and AD groups (Fig 2D). Again, our previously published results for 12 specimens also measured by the Illumina Infinium HD Methylation Assay [10] were positively correlated with those from MS-HRM (*r* = 0.77, *P* = 0.003**, **P < 0.01; Pearson correlation coefficients) (Fig 2E).

To examine the use of methylation levels in the *COASY* gene promoter region for the diagnosis of aMCI and AD, subjects were divided into 2 groups, aMCI + AD and NC, and receiver operating characteristic (ROC) analyses were performed. Cutoff values for the ROC analyses were set as optimal thresholds that would achieve the best combination of sensitivity and specificity. The results suggested that diagnosis was possible with high accuracy (Area under the curve (AUC): 0.99, 95% confidence interval (CI): 0.97–1.01, *P* < 0.0001, sensitivity 96.6%, specificity 96.7%, cutoff value 31.8) (Fig 2C). Similar results were obtained for comparisons of NC vs. aMCI and NC vs. AD (NC vs. aMCI, AUC: 1.00, 95% CI: 0.99–1.00, *P* < 0.0001, sensitivity 100%, specificity 96.7%, cutoff value 31.8; NC vs AD, AUC: 0.98, 95% CI: 0.95–1.01, *P* < 0.0001, sensitivity 93.3%, specificity 96.7%, cutoff value 31.9).

In the case of methylation levels in the *SPINT1* gene promoter region, the results of ROC analyses suggested that aMCI and AD could be diagnosed with moderate accuracy (AUC: 0.78, 95% CI: 0.69–0.88, *P* < 0.0001, sensitivity 63.8%, specificity 83.3%, cutoff value 39.0) (Fig 2F). Again, similar results were obtained for comparisons of NC vs. aMCI and NC vs. AD (NC vs, aMCI, AUC: 0.79, 95% CI: 0.67–0.90, *P* < 0.0005, sensitivity 60.7%, specificity 83.3%, cutoff value 39.0; NC vs. AD, AUC: 0.78, 95% CI: 0.66–0.90, *P* < 0.0005, sensitivity 66.7%, specificity 83.3%, cutoff value 40.0).

### Associations between *COASY* gene promoter region methylation levels and background characteristics

A positive correlation was observed between *COASY* gene promoter region methylation level and CDR-SB in AD subjects. In the NC, aMCI and AD groups, age and methylation level were not associated. Also, methylation level did not differ with gender or presence of *APOE* allele 4. In the aMCI and AD groups, no associations were observed between methylation levels and duration of disease, age of onset, MMSE or BEHAVE-AD. Furthermore, methylation levels did not differ with presence of HSV-1 antibody (Table 2).

**Table 2:** Comparisons between individual background characteristics and *COASY* promoter region DNA methylation levels.

*COASY* methylation level vs	NC (n = 30)	MCI (n = 28)	AD (n = 30)	All subjects (n = 88)
Age (years)	*ρ* = -0.161, *P* = 0.396	*ρ* = 0.169, *P* = 0.391	*ρ* = -0.043, *P* = 0.821	*ρ* = 0.076, *P* = 0.484
Sex	*P* = 0.152	*P* = 0.520	*P* = 0.883	*P* = 0.973
Duration of disease (months)	-	*ρ* = -0.289, *P* = 0.136	*ρ* = 0.242, *P* = 0.198	*ρ* = -0.014, *P* = 0.484
Age at onset (years)	-	*ρ* = 0.196, *P* = 0.317	*ρ* = -0.152, *P* = 0.422	*ρ* = -0.043, *P* = 0.751
MMSE score	-	*ρ* = -0.049, *P* = 0.805	*ρ* = -0.165, *P* = 0.385	*ρ* = -0.046, *P* = 0.732
BEHAVE-AD	-	*ρ* = -0.063, *P* = 0.750	*ρ* = 0.074, *P* = 0.698	*ρ* = -0.014, *P* = 0.915
CDR-SB	-	*ρ* = -0.020, *P* = 0.920	*ρ* = 0.529, *P* = 0.003 **	*ρ* = 0.151, *P* = 0.258
*APOE* ε4 carrier	*P* = 0.646	*P* = 0.194	*P* = 0.791	*P* = 0.184
HSV-1 carrier	-	*P* = 0.583 ^a^	*P* = 0.804 ^b^	*P* = 0.691 ^c^

Associations were examined by Spearman’s rank correlation coefficients, except for sex, *APOE* ε4 carrier, and HSV-1 carrier. For these parameters, groups were compared using Student’s t-test.

** *P* < 0.01

^a^ n = 27

^b^ n = 28

^c^ n = 55.

### Associations between *SPINT1* gene promoter region methylation levels and background characteristics

In NCs, methylation levels in the *SPINT1* gene promoter region were negatively correlated with age. There was also a negative correlation between methylation levels and CDR-SB in aMCI subjects. In the AD group, methylation levels were high for subjects positive for anti-HSV-1 antibodies. However, methylation levels did not differ with sex or presence of *APOE* allele 4. In the aMCI and AD groups, methylation levels were not correlated with duration of disease, age at onset, MMSE or BEHAVE-AD (Table 3).

**Table 3:** Comparisons between individual background characteristics and *SPINT1* promoter region DNA methylation levels.

*SPINT1* methylation level vs	NC (n = 30)	MCI (n = 28)	AD (n = 30)	All subjects (n = 88)
Age (years)	*ρ* = -0.489, *P* = 0.006 **	*ρ* = 0.271, *P* = 0.164	*ρ* = -0.317, *P* = 0.088	*ρ* = -0.097, *P* = 0.370
Sex	*P* = 0.704	*P* = 0.762	*P* = 0.985	*P* = 0.775
Duration of disease (months)	-	*ρ* = 0.063, *P* = 0.749	*ρ* = -0.123, *P* = 0.517	*ρ* = -0.038, *P* = 0.777
Age at onset (years)	-	*ρ* = 0.181, *P* = 0.357	*ρ* = -0.242, *P* = 0.197	*ρ* = -0.014, *P* = 0.916
MMSE score	-	*ρ* = 0.029, *P* = 0.883	*ρ* = 0.256, *P* = 0.173	*ρ* = -0.082, *P* = 0.541
BEHAVE-AD	-	*ρ* = -0.075, *P* = 0.703	*ρ* = -0.116, *P* = 0.543	*ρ* = -0.148, *P* = 0.268
CDR-SB	-	*ρ* = -0.414, *P* = 0.029 *	*ρ* = -0.069, *P* = 0.718	*ρ* = -0.148, *P* = 0.268
*APOE* ε4 carrier	*P* = 0.986	*P* = 0.186	*P* = 0.429	*P* = 0.109
HSV-1 carrier	-	*P* = 0.474 ^a^	*P* = 0.014 * ^b^	*P* = 0.024 * ^c^

Correlations were examined by Spearman’s rank correlation coefficients, except for sex, *APOE* ε4 carrier and HSV-1 carrier, for which groups were compared using Student’s t-test.

* *P* < 0.05

** *P* < 0.01

^a^ n = 27

^b^ n = 28

^c^ n = 55.

In addition, a positive correlation was observed between DNA methylation levels in the *COASY* and *SPINT1* gene promoter regions (*r* = 0.542, *P* = 0.000****, ****P < 0.0001; Pearson correlation coefficients).

## Discussion

In the present study, we measured DNA methylation by MS-HRM in the promoter regions of the *COASY* and *SPINT1* genes, which the results of previous screening had indicated as candidate genes. Among them, we demonstrated that, in aMCI and AD patients, methylation levels in the promoter regions of the *COASY* and *SPINT1* genes were higher than in NCs (Fig 2A and 2D). There was no difference in methylation levels between aMCI and AD subjects suggesting a common molecular basis for changes in the methylation of these genes. Thus, while changes in both could be involved in neurodegeneration, for instance, they would not be a sensitive severity marker that could rigorously differentiate between aMCI and AD.

The present research is an extension of our previous study. However, its use of MS-HRM instead of pyrosequencing to measure DNA methylation levels afforded measurement of DNA methylation levels over a wider area. This clearly showed that DNA methylation levels in the *COASY* and *SPINT1* regions were elevated in the patient group (aMCI and AD) as compared to NCs, in contrast to our previous finding for the *NCAPH2/LMF2* regions.

In PCR for the analysis of the gene promoter regions in this study, although amplification bias could be a problem, as there was a correlation between the results of MS-HRM and those from the Illumina Infinium HD Methylation Assay, we considered our findings to be reasonable (Fig 2B and 2E).

The targets of MS-HRM conducted in the present study were a 264 bp area of the *COASY* promoter region including 14 CpG and a 224 bp area of the *SPINT1* promoter region including 12 CpG. As these areas have a wider range than those usually analyzed by pyrosequencing, we considered this technique to have high detection capability. The results suggested that elevated DNA methylation levels in the *COASY* and *SPINT1* promoter regions, particularly in the former, would be a useful common biomarker for the diagnosis of aMCI and AD.

In the comparison of the aMCI and AD groups, there were no differences in methylation levels in the *COASY* and *SPINT1* gene promoter regions and there was no correlation with MMSE (Table 2). However, for AD, methylation levels in the *COASY* gene promoter region were correlated with CDR-SB, an indicator of severity, suggesting the possibility that DNA methylation progresses as severity increases. The reason for this discrepancy is considered to be because CDR-SB is a more sensitive indicator of dementia severity. Based on the MMSE and CDR-SB scores, disease severity seemed relatively mild in our AD subjects (Table 1) so if many more patients with moderate or more severe dementia were included, we might see a difference in DNA methylation levels between aMCI and AD. A possible reason for the lack of a correlation in aMCI was thought to be the small range of CDR-SB in these subjects.

Regarding DNA methylation in the *SPINT1* gene promoter region, there was a negative correlation with CDR-SB for aMCI. There was also a negative correlation with age (Table 3). While the reasons for this are unclear, intergroup differences in DNA methylation levels were smaller in the *SPINT1* gene promoter region than in the *COASY* gene promoter region, suggesting that DNA methylation levels are controlled by various factors, and are influenced by age and infection in particular. In previous research, it was reported that DNA methylation changed in pace with age [27] and that hypomethylation was occurring in *PASG* mutant mice, a model of aging [28]. However, the results of the present study suggested that there is no uniform pattern of changes in methylation due to aging; it varies with the gene. Furthermore, DNA methylation levels were high in the *SPINT1* gene promoter region in subjects infected with HSV-1 (Table 3), suggesting that HSV-1 infection could be a cause of DNA methylation. However, we do not know whether HSV-1 infection exerts an influence on DNA methylation in NCs and this is a limitation of our study.

Although *APOE* allele 4 is known to be a genetic risk factor of AD, it was not involved in DNA methylation in the *COASY* and *SPINT1* gene promoter regions (Tables 2 and 3). In addition, there was a positive correlation between DNA methylation levels in the *COASY* and *SPINT1* gene promoter regions, again suggesting the existence of a common molecular basis for the DNA methylation that occurs in these regions.

In the present study, changes in DNA methylation levels in the *COASY* and *SPINT1* gene promoter regions were those in the blood and not a direct indication of changes in the brain. However, as a previous study had demonstrated similar alterations in DNA methylation in the blood and in the brain [29], we speculated that changes in methylation of blood DNA would reflect changes in brain DNA methylation levels, though it is unclear whether this would apply to the *COASY* and *SPINT1* genes, which is another limitation of the present study. Also, even if we assume that changes in DNA methylation levels in the *COASY* and *SPINT1* gene promoter regions did not directly reflect gene expression in the brain, based on the findings of other studies, their methylation levels would still potentially be a useful biomarker [30, 31].

Encoding for coenzyme A (CoA) synthase, the *COASY* gene is involved in the biosynthesis of CoA from pantothenic acid. Regarding mutations in the *COASY* gene, one is known to be associated with neurodegeneration with brain iron accumulation (NBIA) [32]. In AD research, it has been reported that *COASY* single nucleotide polymorphisms (SNPs) are a risk for developing AD in Down Syndrome patients [33] and it has been observed that cholinergic dysfunction in AD is due to reduced production of acetyl CoA [34]. Further, as it has been suggested that decreased expression of CoA synthase due to DNA methylation is involved in the mechanism for the latter, we could hypothesize that changes in methylation similar to those in blood DNA also occur in brain DNA.

Encoding for serine protease inhibitor Kunitz type 1, the *SPINT1* gene is also known by its other name of hepatocyte growth factor inhibitor-1 (HAI-1). Binding with hepatocyte growth factor activator (HGFA), it controls the proteolytic activation of HGF [35]. It has been reported that these substances are also produced by astrocytes and that protein expression of *SPINT1* is weak in the AD brain [36] so if we postulate that changes in DNA methylation levels similar to those in the blood also occur in the brain, this would be consistent with the increased methylation in the *SPINT1* gene promoter region of AD subjects in the present study.

White blood samples show heterogeneity and it has been reported that there is a difference between polymorphonuclear cells and mononuclear cells regarding DNA methylation levels [37, 38]. However, regarding differential white blood cell counts, as the blood samples used in the present study produced no abnormal results and there were no deviations among them, we considered that the influence of this on *COASY* and *SPINT1* gene promoter methylation levels to be small.

Another limitation of the present study is that, as it did not analyze gene expression in the blood, the effect of changes in methylation levels in promoter regions on gene expression is unknown. Thus it is also unknown whether changes in methylation are directly involved in the mechanism of onset of AD. However, based on our examination of the functions of the *COASY* and *SPINT1* genes as well as the fact that the present study analyzed regions including several CpGs rather than individual CpGs, it was considered possible that these genes are a causative influence in AD. Also, at minimum, we consider that changes in methylation levels would be a useful diagnosis biomarker.

As MS-HRM was able to cover several CpGs over a wider area with one set of primer, we considered it to have the advantages of being more sensitive and affordable. However, although a similar pattern of methylation has been reported in neighboring CpG sites [39], we feel that not clarifying the methylation status of individual CpGs by bisulfite sequencing and pyrosequencing is a limitation of our study. There could be a pattern for the methylation statuses of individual CpGs [30, 31] so it will be necessary to investigate the *COASY* and *SPINT1* promoter regions in more detail to see if such a pattern exists and identify individual CpGs specific to aMCI and AD.

While our analysis only focused on changes in DNA methylation in promoter regions, changes in DNA methylation were also occurring in gene bodies and other non-promoter regions in AD and aMCI [10]. However, many aspects of the significance of such changes to aMCI and AD remain unclear [40] and should be clarified in the future in view of their importance.

Also, recent research has suggested the possibility of using methylation patterns in dead cell-derived cell free circulating DNA (cfcDNA) in plasma and serum as a new diagnostic biomarker [41] in relation to traumatic or ischemic brain damage. Although there is no direct association between ischemic damage and the onset of aMCI or AD, future research should investigate cfcDNA in aMCI and AD to determine if the methylation of such DNA has a common molecular basis with the DNA methylation reported in this study and further clarify the molecular mechanism by which DNA methylation occurs.

Our study demonstrated that DNA methylation was increased in the *COASY* and *SPINT1* gene promoter regions in aMCI and AD. This indicates the possibility that it would be a useful diagnosis biomarker. It is also possible that our findings will lead to the elucidation of a new mechanism of AD onset.
